# A Day with Gold Miners in the Amazon Fighting against Malaria

**DOI:** 10.4269/ajtmh.24-0687

**Published:** 2025-01-21

**Authors:** Maylis Douine

**Affiliations:** Centre d’Investigation Clinique Antilles–Guyane Inserm1424, Centre Hospitalier de Cayenne, Cayenne, French Guiana

Passing the plunging roots of a mangrove tree, the pirogue glides slowly over the calm water of the river before coming to a halt on the bank. The sun’s rays seeping through the branches are not yet strong in the fresh morning air. I straighten up, a little unsteady on the small wooden pirogue, before descending on the river bank. A few meters farther on, a banner bearing the inscription “Aqui venha receber um Malakit” indicates the presence of the health facilitators. They are sitting underneath a tarpaulin strung between two wooden shacks, one selling equipment for artisanal gold mining and the other buying the gold nuggets extracted from the ground.

In front of the small plastic table, a gold miner with a weathered face and mud-stained pants rolled up over rubber boots is deep in conversation with one of the project’s mediators. The man explains his situation in colloquial Portuguese, his voice shy. I briefly greet them and stop short so as not to interrupt. “I’ve been working on the gold mine for 10 years. I come from a poor family, and in Brazil, where I’m from, there was no work. I came here to earn a little money so that my children could study. But then, there was a military operation, and I had to flee the mine. It’s hard, washing off the mud with a water hose all day, clearing away the stones. My body is bruised. And there are so many diseases, especially malaria. I’ve heard about Malakits, and I want one.”

The health facilitator then kindly explains what a Malakit is. The Malakit is a portable plastic pouch that allows gold miners to diagnose themselves if they have any symptoms of malaria using rapid diagnostic tests. If the test is positive, the kit also contains a full course of antimalarial treatment. However, to obtain a kit, the gold miner must undergo a short training course and demonstrate that he understands how to perform a diagnostic test. The rugged man, used to the harshness of the mine and carrying dozens of kilos of equipment on his shoulders through the jungle, now freezes: “Me, prick myself?,” he asks, clearly reluctant.

I watch the scene, both amused and moved. This man, confronted daily with the dangers of the forest, snakes, and diseases, is frightened by the idea of a simple finger prick. The health facilitator smiles, approaches, and does his best to lighten the mood. “Don’t worry,” he says. “It’s easier than you think. I’ll show you,” he offers, holding up an explanatory video.

The scene that unfolds before my eyes tells me that the months, even years of advocacy and relentless efforts to implement this strategy have served a purpose. I remember the many meetings, the negotiations with national and international authorities, and the debates where we had to defend this new strategy. At the start of the project, many people thought that uneducated people living in precarious conditions would be incapable of carrying out a diagnostic test for themselves or that they would sell the kit to buy alcohol. Such an infantilizing vision. Yet, the idea seemed brilliant; to reach the most vulnerable populations, we had to innovate. There was simply no other option. Malaria wreaks havoc in the clandestine gold mining areas of French Guiana, where access to health care is virtually impossible. After a great deal of thought, we came up with “Malakit,” a simple and effective tool to enable gold miners to take charge of their own health, and it works! All of the tools used to evaluate the system show that the kit is much appreciated by gold miners, that they use it correctly, and that the impact on the spread of malaria is tangible. Thousands of people have been treated thanks to the kit.

I continue to observe the facilitator, admiring his patience. He takes the time to explain the right gestures to the gold miner, showing him how to use the test with drawings and videos on a tablet. This facilitator is a former gold miner himself without a diploma, but he has been able to improve his skills thanks to the project. Today, he is respected within his community, and his pride is visible in every gesture he makes. I make sure that he has all of the necessary equipment, kits, rapid tests, medicines, and training materials he needs. I explore his feelings, challenges, and needs, particularly regarding his life in an isolated environment. This is also the opportunity to do a quick refresher; training gold miners is no easy task, and it is important to ensure that the facilitators remember the necessary methods and skills for the role. “It’s thanks to you that all this is possible,” I tell him at the end of our discussion. “You are essential to this project.” He smiles modestly, but it is certain that without him and the other facilitators, this project would never have seen the light of day. These men and women are the pillars on which our entire system rests. Moral and financial considerations and support for their activities are vital to the success of the strategy.

The Malakit project takes place at the interface between three countries: French Guiana, Suriname, and Brazil. Each of these territories has its own politics, its own challenges, its own language, and its own institutions. Governments change, laws evolve, and stakeholders mutate; so, there is a constant need to adapt. This heterogeneous context makes cooperation difficult but feasible. I think back to the headache of coordinating meetings in four languages to find a venue relatively accessible to all despite bad roads and precarious airlines. It was complex, but it was doable and so rewarding! Today, the challenge is to ensure its sustainability and integration into the health care system. Here, the regulatory challenges were numerous. However, a new malaria epidemic in 2023 and 2024 forced the French authorities to grant exemptions to implement Malakit in French Guiana. The project has now been integrated into the public health programs of Suriname and France, and it is well on the way to being adopted in Brazil. It is such a relief, and it makes me so proud; all of these efforts have not been in vain, and the Malakit strategy is sustainable beyond the end of the research!

But, I pause my thoughts and now realize that a crowd has formed around the health facilitators. This happens regularly; once the first person has been trained, other curious people approach, and the interest snowballs! Despite the rising heat under the tarpaulin, discussions are flowing. One very talkative young woman, her long black hair flowing down her back, is making fun of a young man who says he does not need the kit because he has never had malaria: “And you think you’re stronger than everyone else? What are you going to do when you’re sick in the middle of the jungle? You’ll be very happy to be able to treat yourself with good quality and free medication!”

Noticing me, she approaches me. I concentrate on understanding her in Portuguese, despite the noise of the pirogue engines and generators. “I’ve heard that you and your team invented this kit? Is it true that it only exists here?” I confirm. “It should be distributed all over the world; it’s really useful!”

I think of the colleagues in other parts of the world who have contacted me.

Indeed, this strategy embodied by this small waterproof fabric pouch—which can be locally produced, such as with our charming leafy print—has the potential to address the needs of various hard-to-reach populations worldwide. However, securing funding will remain a significant challenge.

I turn around one last time before getting back on the pirogue. Yes, their activity is destructive to the environment. But, at the same time, it is all of us who buy the gold and give it its value! We have a responsibility to the health of the people who work in this hostile and fascinating jungle.

